# Eating Disorders in Pregnant and Breastfeeding Women: A Systematic Review

**DOI:** 10.3390/medicina56070352

**Published:** 2020-07-15

**Authors:** María Martínez-Olcina, Jacobo A. Rubio-Arias, Cristina Reche-García, Belén Leyva-Vela, María Hernández-García, Juan José Hernández-Morante, Alejandro Martínez-Rodríguez

**Affiliations:** 1 Faculty of Health Sciences, University of Alicante, 03690 Alicante, Spain; mmo36@alu.ua.es (M.M.-O.); mhg30@alu.ua.es (M.H.-G.); 2 LFE Research Group, Department of Health and Human Performance, Faculty of Physical Activity and Sport Science-INEF, Polytechnic University of Madrid, 28040 Madrid, Spain; ja.rubio@upm.es; 3 Faculty of Nursing, San Antonio Catholic University of Murcia, 30107 Murcia, Spain; jjhernandez@ucam.edu; 4 Department of Health, Vinalopó University Hospital, 03293 Elche, Spain; bmleyva@vinaloposalud.com; 5 Department of Analytical Chemistry, Nutrition and Food Science, Faculty of Sciences, University of Alicante, 03690 Alicante, Spain; amartinezrodriguez@ua.es; 6 Alicante Institute for Health and Biomedical Research (ISABIAL Foundation), 03010 Alicante, Spain

**Keywords:** eating disorder, feeding, psychology, pregnancy

## Abstract

*Background and objectives:* Pregnancy is a stage associated with various biopsychosocial changes. These changes, along with concerns about keeping an adequate weight, can modulate an individual’s risk for psychological disorders, especially eating disorders (EDs). The aim of this review was to investigate the prevalence, associated risks, and consequences of eating disorders in pregnancy and in breastfeeding mothers. *Materials and Methods:* A systematic review was carried out following the PRISMA guidelines in the scientific databases: PubMed, Web of Science, Scopus, and PsycINFO. Search terms related to EDs, pregnancy, and breastfeeding were used. The evaluation of the methodological quality of the studies was carried out using different scales; CASP (Checklist for Cohort Study), NICE (Methodology Checklist for Cohort Study), ARHQ (Methodology Checklist for Cross-Sectional), and NOS (Newcastle-Ottawa Scale for Cohort). *Results:* From 2920 studies, 16 were selected to study EDs in pregnant women and 2 studies in nursing mothers. Most of the studies used questionnaires and scales as tools for the diagnosis of EDs. Binge eating, anxiety, and depression were the most common comorbidities of EDs, accompanied in most cases by excessive concern about weight gain. The consequences of EDs are diverse. The prevalence of EDs in this population is estimated to be 1 out of 20. *Conclusions:* Eating disorders are related to anxiety and depression and have negative consequences for both mothers and fetuses (cesarean, miscarriages, premature births). More research on the field to determine the risk factors for EDs in the population of pregnant and lactating women is needed.

## 1. Introduction

Pregnancy is a time of change for the maternal organism since it needs to adapt to different biopsychosocial modifications, body composition and nervous system, eating habits, and physical activity, among others [1]. All of these changes can contribute to certain complications and risks of psychiatric disorders in a pregnant woman [2].

Maternal psychological distress is accompanied by metabolic and functional changes that may also influence fetal development. These variations include autonomic changes, disturbance of maternal circadian rhythms, and behavioral changes that may influence maternal diet and lifestyle [3]. Maternal psychological distress is associated with high maternal stress hormones such as cortisol, adrenocorticotropic hormone (ACTH), and adrenaline [4].

At present, eating disorders (EDs) are a health problem with a high impact on society [5]. Community-based studies are important because only a minority of individuals suffering from eating disorders enter treatment. Among European women, the prevalence of anorexia nervosa is <1–4%, bulimia nervosa <1–2%, binge eating disorder 1–4%, and subthreshold eating disorders 2–3% with considerable variation by area, age group, and ethnic origin [6]

During pregnancy, 21.7% of women suffer from depression [7]. This is associated with adverse fetal, obstetric, and neonatal outcomes and is a risk factor for postpartum depression. One of the factors that can condition the depressive picture is insomnia [8]. Fifty percent of women present insomnia during pregnancy and ten percent before pregnancy. Pregnant women see their quality of sleep deteriorate and the number of hours of sleep also decreases, particularly in the third trimester of pregnancy, so attention should be paid to this conditioning factor [9,10,11].

In the postpartum period, dissatisfaction with body weight and shape are normative, even in women without EDs. In the first month after delivery, 75% of women are concerned with weight retention, and by four months postpartum, 70% of women are attempting to lose weight [12]. During this time, women report an increasing food restraint, such as food avoidance and/or adherence to specific food rules [12]. Sex hormones also influence the type of developed ED. Estrogen is known to stimulate hypothalamic pituitary adrenal (HPA) activity, thereby increasing stress responsivity [13]. Androgens, in contrast, tend to reduce HPA activity and thereby reduce stress responsivity [13].

Research suggests that up to 7.5 per cent of pregnant women suffer from an eating disorder [14], which is a severe psychiatric illness, linked to a distorted perception of their own body and dissatisfaction, characterized by marked behavioral changes and an excessive concern with body weight and/or shape. These illnesses can become chronic and debilitating and are associated with significantly increased mortality rates [15]. The most prevalent EDs are anorexia nervosa (AN), bulimia nervosa (BN), binge eating disorder (BED) [16], and others like dysmorphic muscle disorder, avoidant/restrictive food intake disorder, pica, rumination, atypical AN, sub-threshold BN, sub-threshold binge eating disorder, purging disorder, and night eating syndrome [17].

According to the Diagnostic and Statistical Manual of Mental Disorders (DSM-V-TR), AN is an ED with an adverse reaction to eating due to the pursuit of thinness, the refusal to maintain normal body weight, and the distortion of body image (size and shape) [18]. BN is characterized by repeated episodes of binge eating, followed by inappropriate compensatory methods to prevent weight gain: vomiting, abuse of laxatives, diuretics and other drugs, fasting, and excessive physical activity [19]. BED consists of a large amount of food intake, greater than most people would carry out during that time and circumstances; however, such intake is not followed by compensatory behaviors [20]. Finally, non-specific eating disorder (EDNOS) are usually incomplete tables of AN and BN, with similar symptoms, including the use of compensatory behaviors after the ingest of normal amounts of food or episodes of compulsive eating without compensatory behaviors [21].

The role of food intake, especially micronutrients as vitamins, is relevant during this period, the deficiency of some nutritional factors involved in one-carbon metabolism, such as vitamin B-2, B-6, B-12, choline, betaine, and n-3 polyunsaturated fatty acids, may be also associated with neutral tube defects (NTDs) in babies [22]. In addition, the intake of vitamin D is essential, the need of it could be higher in pregnancy because it was involved in several physiological processes. Although its involvement in bone metabolism and calcium management in bone health processes is widely confirmed, literature repeatedly suggested non-musculoskeletal targets such as the immune system, regulation of cell proliferation and differentiation, and glucose metabolism [23].

EDs patients are difficult to treat, have low recovery rates with a high risk of relapse. EDs are associated with a low quality of life, high rates of psychosocial comorbidity, and risk of premature mortality [16]. In this regard, there is evidence from extensive cohort studies and registration data showing that EDs and weight fluctuations have adverse effects on the course of pregnancy and birth outcomes [24]. In addition, research indicates that some women have unrealistic expectations about their bodies in the postpartum period, so physical changes can produce body image variations, leading to a degree of dissatisfaction [25]. These disorders are associated with metabolic and endocrine diseases, psychological and nutritional changes that have negative effects on both mothers and fetus, including a high prevalence of abortions, low birth weight, and complications of postpartum depression [17]. Evidence shows that diet is related to inflammation, oxidative stress, and brain function and plasticity, which are factors potentially involved with mental disorders [17].

However, the information in the literature is dispersed, therefore the intention is to gather the information of the last decade in a single study. There are no studies in the literature that determine the effects, prevalence, risks, and consequences of individual EDs in pregnant and breastfeeding women. For this reason, the present systematic review aims to provide a synthesis of evidence regarding the effects, associated risks, and consequences of EDs in both pregnancy and breastfeeding.

## 2. Materials and Methods

### 2.1. Design

This literature review was conducted using the informative guidelines for systematic reviews [26]. It was designed following the recommendations of the PRISMA Statement [27].

### 2.2. Eligibility Criteria

Inclusion criteria: The selection protocol was developed based on the population, intervention, comparison, and outcome (PICO) questions. Any observational study that recruited pregnant or lactating women (Population) and that analyzed the presence and impact of EDs (Outcome) on ill mothers versus healthy mothers (Comparison), was chosen for inclusion in the systematic review. The presence or absence of ED should be measured by questionnaires, structured, semi-structured, or scaled interviews. The sample data were pregnant or breastfeeding population with EDs specifically; AN, BN, BED, and EDNOS, included in studies from 2009 to February 2019.

Exclusion criteria: All articles on ED with adolescent or not pregnant-lactating women as the study population were excluded. Moreover, articles with other pathologies (e.g., obesity, diabetes, or respiratory insufficiency) were also excluded as well as the ones that concerned children of mothers with EDs, but not mothers themselves.

### 2.3. Search Strategy

A search strategy was conducted to identify studies that worked with or evaluated pregnant and lactating women diagnosed with ED. The following databases were searched: Pubmed, Web of Science, Scopus, PsycINFO. In PubMed, the following Boolean descriptors and operators were combined: (pregnant or pregnancy or breastfeeding) and (“eating disorders” or anorexia or bulimia or “binge eating disorder”). This strategy was adapted for each database. All articles with the established search equation were obtained, and duplicates were eliminated.

### 2.4. Data Collection

A critical reading of the documents was carried out to confirm the validity of the studies and check that they answered the research question. In addition, the design and sample were correct, and there were no variables, characteristics, or interests that could influence the interpretations and conclusions. The search was performed independent by two authors (M.M. and M.H.) and collected in a reference manager.

### 2.5. Data Synthesis

The data were extracted. The extraction was done by two researchers independently (M.M., M.H.), and the results were compared. A third author (A.M.) resolved the discrepancies. The aim was to collect the most relevant information from each article included. A previously prepared datasheet included the following variables: name of the main author, year of publication, type of study, journal in which it was published, the population in which it was carried out, intervention, objective, relevant results, tests used, covariates, and hypotheses of the authors.

### 2.6. Methodological Quality

Methodological quality analysis was conducted independently on all full-text papers that met eligibility criteria by two authors (M.M. and M.H.). The main tools were used according to the type of study [28]. As the articles included in this review are analytical, cohort, or cross-sectional studies, the scales used were: the CASP checklist (Checklist for Cohort Study) [28], the NICE checklist (Methodology Checklist for Cohort Study) [28], and the NOS scale (Newcastle-Ottawa Scale) [28]. The results of this analysis are available in the appendix to the main document (Tables S1–S3). Studies that were judged as “Poor” by both authors were excluded from this review.

## 3. Results

### 3.1. Study Selection

**Figure 1:**
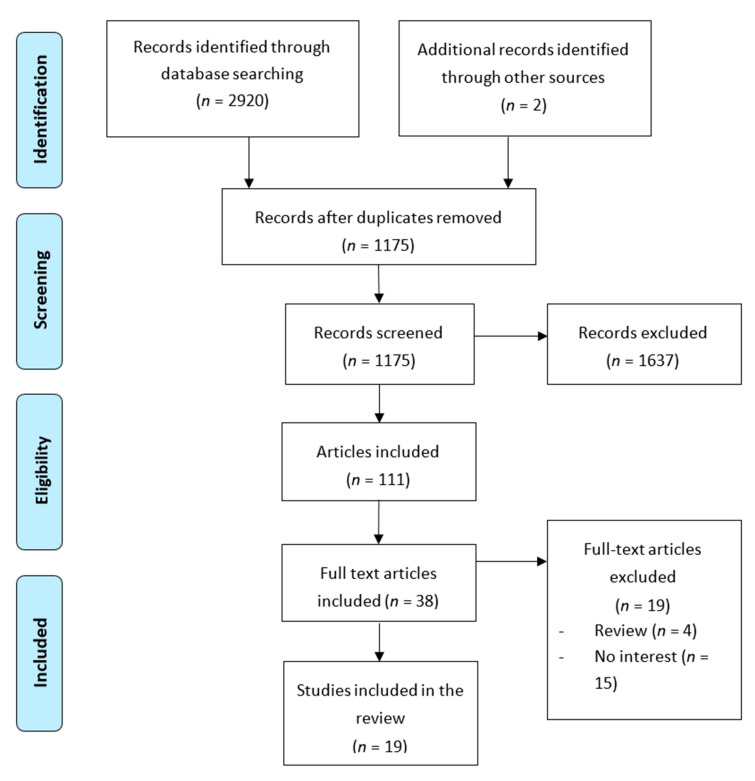
Flow chart for study selection process.

A search of databases resulted in a total of 2920 articles. Figure 1 summarizes the flow of studies meeting criteria of final inclusion. Additional information on the reason for exclusion can be found for each of the articles studied. However, the main reasons were that they were narrative reviews or that they did not analyze variables of interest. Eighteen studies were included in the final review, 17 cohorts and one cross-sectional. Seven papers are based on the Norwegian Mother and Child Cohort Study (MoBa) [12,29,30,31,32,33,34], two on data from pregnant women from the Avon Longitudinal Study of Parents and Children (ALSPAC) [35,36], three on pregnant women of the ECCAGE (The Study of Food Intake and Eating Behaviors in Pregnancy) [37,38,39], two on women from the Nutrition and Stress in Pregnancy (NEST-p) study [40,41], one on the Netherlands population cohort of the Generation R Study [42], and the remaining three studies on the pregnant women’s information from different clinics in Hong Kong [33], Stockholm [43], and London [14].

### 3.2. Study Description

Characteristics of studies, including details of the sample and size, measures used to assess the disorders, and significant findings are detailed in Table 1.

### 3.3. Synthesis of Findings

In all studies, the diagnosis of ED was based on the criteria of the Diagnostic and Statistical Manual of Mental Disorders-IV (DSM-IV). In AN, since they are pregnant women, the criterion of amenorrhea was not used. The information to establish the diagnosis of ED, changes in weight, women’s perceptions, feelings, food consumption and behavior, was obtained using different scales and questionnaires. In the studies based on the MoBa, women completed the Medical Birth Registry of Norway (MBRN), the Hopkins Symptom Checklist-25 [72], and six different questionnaires at different times and on different topics [51]. In the ALSPAC study, information came from questionnaires on sociodemographic, anthropometric, and fertility variables at different time points, in addition to the consumption frequency questionnaire (FFQ) [54]. While in the ECCAGE study, they used their own questionnaire and the EDE-Q [47]. The study of Women in the Nutrition and Stress in pregnancy (NEST-p) completed the Structured Clinical Interview for Axis I DSM-IV-TR disorders (SCID-I) [59], the questionnaire to examine eating disorders (EDE-Q), the Spielberger State Anxiety Inventory (STAI) [60], the Perceived Stress Scale (PSS) inventory [62], and the Pregnancy-Related Anxiety Questionnaire (PRAQ-R) [63]. In the remaining studies, the EDE-Q, FFQ, EDDS, and postnatal questionnaires on breastfeeding were used differently.

#### 3.3.1. Concern about Weight and Other Symptoms 

In different studies, it was observed that there was a concern about weight during the pregnancy period (40.2% of pregnant women) [29,34,37,39]. Together with episodes of binge eating (17.3%) [37], symptoms of anxiety and depression [65] were the most frequent symptoms of ED. Such concern about weight in mothers with EDs caused them to engage in inappropriate behaviors such as self-induced vomiting or misuse of diuretics [37]. The prevalence of EDs during pregnancy is: 0.5% AN, 0.1% BN, and 1.8% BED, 0.1% used purging and 5% EDNOS [14], approximately 5.1–7.5% of women during pregnancy.

It was observed that mothers with ED had greater weight gain during pregnancy than healthy mothers [9,19,20,29,73,74]. There is a controversy about weight loss, with research showing greater decreases during the first six months after childbirth in mothers with ED [9,19,20,29,73,74], while others claim that postpartum weight retention is greater than in healthy controls [39]. Weight gain can be positive for those suffering from AN, as it can mitigate the adverse effects of this disorder [34] and protect the demands of the developing fetus. In turn, weight gain in mothers with BN and BED seems to be related to food intake [31,52,54] resulting in excessive gain [36]. This excess weight may also be due to the higher prevalence of binge eating in mothers because of anxiety symptoms [42].

#### 3.3.2. Effects and Complications of EDs during Pregnancy 

Pregnancy could have positive effect in women with eating disorders [32,39,56]. Women with BN have shown a reduction of symptoms and restrictive episodes [8,54]. However, these symptoms may even increase afterwards, at the postpartum and breastfeeding period [32,39,41]. Others state that the presence of ED is higher during and especially after birth [43], finding it difficult to balance the desire to restrict caloric intake with the impulses to eat [43]. So, gestation may be a period of greater vulnerability. As for cortisol hormone, different patterns of circadian salivary cortisol were observed in women with active ED during pregnancy [31]. Specifically, low morning cortisol levels were apparent in women with active ED during pregnancy, compared to women who had recovered from an ED before pregnancy and women without ED. Each subtype has important medical complications. Associated with AN are weight loss and malnutrition. BN purging behaviors lead to hydroelectric imbalance. Binge eating disorders are related to obesity, both during pregnancy and in the postpartum period, because of excessive gain and retention [38].

#### 3.3.3. Feeding during Pregnancy and Cessation of Breastfeeding

The quality of the diet has also been investigated [42,56] by analyzing the dietary intake during pregnancy of women with and without ED. It was observed that mothers with ED consumed less meat in favor of products such as soy and legumes. They obtained higher scores on the ‘vegetarian’ dietary pattern. They were also less likely to consume butter, whole milk, sugars, and saturated fats. Their intake of macronutrients, vitamins, and minerals was correct. However, they were more likely to consume caffeine (>2500 mg caffeine/week). Both maternal diet and nutritional intake influence fetal development [44]. With breastfeeding, it has been concluded that there were no differences between mothers with ED and healthy mothers concerning the initiation and cessation of breastfeeding [41]. However, it seems that in the case of AN and EDNOS, the risk of abandonment is higher [30].

## 4. Discussion

This review aimed to investigate the prevalence, obstetric and nutritional problems associated with EDs in pregnant and breastfeeding mothers and the consequences for their babies. ED typically affects women of reproductive age [75]. Several epidemiological studies [58,76,77] show that approximately 1 out of 20 (5.1–7.5% of women during pregnancy), 88 women may experience some ED during pregnancy. In line with other reviews [78], there is sufficient evidence to determine that EDs are associated with anxiety and depressive symptoms during pregnancy [37]. In addition, these symptoms, along with stress, are associated with elevated levels of cortisol [31].

During the postpartum period, depression can lead mothers to be unresponsive, inconsistent, or refuse the baby [79]. In the studies analyzed in this review, mothers are not separated by age. However, it is known that adolescent women are more likely to face higher pregnancy-related maternal and perinatal morbidity and mortality than adult women [80]. EDs do not just affect the mother’s health. As has been observed [51,56,62], EDs influence the formation, growth, and birth. Depending on the subtype of ED, the consequences appear to be different. The complications that have been observed with a higher prevalence in women with AN include hypothermia, hypotension, and edema, hypertension, miscarriages, cesarean [65,81], premature births, and reduced intrauterine growth [81]. Women with EDs also have a higher proportion of unwanted pregnancies than healthy mothers [38]. For women with BN, higher proportions of induced abortions [82], increased risk of hyperemesis, babies with microcephaly, and small for gestational age [83] have been reported. In addition, it has been observed [65] that the descendants’ sex may also be modified by ED, with a higher probability of sons rather than daughters for women with BED and lower for those suffering from AN or BN. Furthermore, in the transgenerational study conducted by Hunna J Watson [33], it was observed that utero conditions can determine susceptibility to diseases later in life. Mothers born at a lower birth weight were more likely to develop AN. However, they report that lifetime BN was not associated with perinatal factors.

Coinciding with the results presented [32,33,78], other investigations have also observed that mothers with EDs have a higher risk of cesarean delivery [84,85], have large-for-gestational-age babies, with higher weights [70]. It seems that breastfeeding can be a positive action since, in addition to being beneficial to the baby, it strengthens attachment and psychological outcomes [41]. As for the duration of breastfeeding for mothers with ED, insufficient scientific literature was found. More research would be needed to reach a conclusion. It has been observed that education and income are associated with prolonged breastfeeding [53], which is also related to weight loss after childbirth [63,79]. In addition, a smaller population-based study in Sweden found that, compared to women without EDs, women grouped by prior or current ED were significantly more likely to have terminated breastfeeding by three months postpartum [86]. On the same direction, the ALSPAC study found that women with EDs were significantly less likely to cease breastfeeding relative to referent women [35,87].

There are studies about the intake of nutrients, including caffeine, in pregnant mothers with and without ED [85,86]. This substance has been found to cross the placenta and may have some negative effects on the fetus [82]. Caffeine consumption recommended by the Food Standards Agency is less than 200 mg/day of caffeine [88]. However, it has been observed [36] that mothers with ED are two times more likely to consume more than 350 mg/day (2500/week) or 25 cups of coffee per week compared to healthy women. Consistent with the suggestions of Abraham et al. [83], this high caffeine consumption could be due to their desire to suppress appetite. 

In line with the analysis of intakes, the study carried out by Nguyen about AN women [42] aimed to evaluate the relationship between diet quality and ED. Like the research carried out by Golding et al. [52], both use the FFQ [54] as a measurement instrument. It only questions the frequency with which certain foods are consumed, but not the amount. It has been observed that women with past or active ED often report difficulties in determining what adequate portion size is. It is possible that for certain foods, women with AN eat smaller portions than women with BN [36]. Therefore, it cannot be claimed that mothers with EDs eat better than healthy mothers, because although the type of food chosen is correct [42], it is not known whether the amount is also adequate.

Maternal malnutrition is a risk factor for maternal, fetal, and neonatal complications. It ranges from undernutrition to over dietary intake before and in the pregnant state [89]. In line with the results obtained in the review, S Triunfo et al. determined that pre-pregnancy underweight and insufficient gestational weight gain, characteristics of AN, are considered as individual risk factors for the spontaneous interruption, preterm birth, fetal growth restriction, and hypertensive disorders, strongly associated with poorer perinatal outcome [89]. If vomiting is considered a compensatory behavior (typical of BN), it may in theory adversely affect appetite, hydration, and nutritional intake; adequate nutrition may already be a challenge for women with EDs [90]. However, all EDs have negative consequences on the mother’s nutritional status and associated problems.

This review has some limitations. Firstly, the results of different studies cannot be generalized. In the studies based on the MoBa sample of the total participants who were invited, only 40% accepted. Although this result does not necessarily imply that the sample is biased [91], the sample of pregnant mothers could represent women with less severe forms of EDs. In addition, even though WHO has described that the weight variations of pregnant mothers are similar in Norway, Brazil, and the United States, it has not been demonstrated that the results obtained in Norwegian mothers can be extrapolated to the general population [92]. Regarding the population of the NEST-p study [58] and the ECCAGE, the samples of BN and BED in the NEST-p were small, the women who remained throughout the study were Caucasian and with a high education level, while the women included in the ECCAGE had low incomes [37,38,39].

Secondly, in some cases, included studies incorporated self-reported body composition data. It may be that some results could have been conditioned in relation to this variable, in terms of the determination or possible presence of an ED [93]. Finally, questionnaires to establish the diagnosis of ED vary from studies. The study carried out with pregnant women in the Hong Kong hospital [65] attempts to establish a relationship according to the trimester of pregnancy and the presence of ED. It concludes that EAT-26 is not an adequate tool since changes in the score between trimesters are not good enough to indicate changes in prevalence [65].

As future lines of research, empirical studies should be carried out to identify both risk and protective factors (family, psychosocial, cultural, eating habits) associated with the development and maintenance of EDs. Furthermore, these should be done in different populations, since it is not possible to extrapolate data because maternal characteristics are not the same according to different geographical areas. Furthermore, it is also necessary that both the diagnostic criteria and the questionnaires used are the same. As noted in another review, it seems that it is necessary specialized treatment, particularly before pregnancy, in relation to eating habits and concerns about weight and body shape.

## 5. Conclusions

About 1 out of 20 pregnant women are at risk for EDs during pregnancy. This situation has negative consequences for both mothers and fetuses (cesarean, miscarriages, premature births). It was observed that there was concern about weight during the pregnancy period (40.2% of pregnant women). Together with episodes of binge eating (17.3%), symptoms of anxiety and depression were the most frequent symptoms of ED. However, it has not been possible to determine exactly which mothers or pregnant woman are a population at risk of presenting these psychological alterations related to eating habits.

## Figures and Tables

**Table 1:** Summary of articles included in the review.

Lead Author, Year	Type of Study	Population Study	Measurement Tools	Results
**Rafael Marques Soares, 2009** [37]	Prospective cohort study of pregnant mothers	Pregnant women who participated in the ECCAGE (The Study of Food Intake and Eating Behaviors in Pregnancy-) [44,45,46]	EDE-Q [47] FFQ [48]	The prevalence of BED in pregnant women is 17.3% (IC 14.5–20). It is higher in those who present anxiety symptoms, depressive symptoms. Inappropriate eating behaviors before pregnancy persisted during pregnancy. A low pre-gestational BMI (<19.8 kg/m^2^) is significantly associated with binge eating during pregnancy. The most common symptoms of EDs during pregnancy are binge eating and weight concerns.
**Cynthia M Bulik, 2009** [49]	Prospective cohort study of pregnant mothers	Pregnant women who participated in the Norwegian Mother and Child Cohort Study (MoBa) [50]	Blood tests Urine analysis MBRN—MoBa MoBa Questionnaire 1 [51] MoBa Questionnaire 4 [51]	Out of 35.929 mothers; 35 reported AN (0.09%), 306 BN (0.85%), 1812 BED (5.04%), and 36 EDNOS (0.1%). In all groups of EDs, the smoking rate is high. Mothers with ED except EDNOS have greater weight gain during pregnancy than healthy mothers. Those with BN or BED, this increase may be related to eating behaviors. Those with AN, gaining adequate weight during pregnancy may mitigate the adverse effects.
**Rebecca A. Swann, 2009** [34]	Prospective cohort study of pregnant mothers	Pregnant women who participated in the Norwegian Mother and Child Cohort Study (MoBa) [50]	Blood tests Urine analysis MBRN—MoBa MoBa Questionnaire 1 [51] MoBa Questionnaire 3 [51] MoBa Questionnaire 4 [51]	Out of 35.929 pregnant mothers; 35 AN (0.09%), 304 BN (0.85%), 36 EDNOS (0.1%), 1812 (5.04%) BED, and 33,742 (93.9%) healthy. The presence of EDs is associated with a concern about weight gain during pregnancy. The increase is greater in those who are concerned about gain than those who are not.
**Leila Torgersen, 2010** [30]	Prospective cohort study of pregnant mothers	Pregnant women who participated in the Norwegian Mother and Child Cohort Study (MoBa) [50]	MBRN—MoBa MoBa Questionnaire 1 [51] MoBa Questionnaire 4 [51]	Of the mothers who started giving BM, 98% did so predominantly, with no difference between mothers with EDs-healthy mothers. At six months, 83% of the mothers were still breastfeeding, but only 15% were predominantly feeding. The risk of abandonment is higher in mothers with AN and EDNOS. There are no significant differences between the early abandonment of mothers with EDs and healthy mothers.
**Anna Maria Siega-Riz PhD, RD, 2011** [31]	Cross-sectional cohort study	Pregnant women who participated in the Norwegian Mother and Child Cohort Study (MoBa) [50]	MBRN—MoBa MoBa Questionnaire 1 [51] MoBa Questionnaire 3 [51] MoBa Questionnaire 4 [51]	The average weight gain of the population was: 2.5 kg at 17–20.1 weeks, 9.3 kg at 27.4–29.7, and 15 kg at delivery. Women with BN and BED gained significantly more weight on average.
**A Easter, 2011** [35]	Prospective longitudinal study of a birth cohort	Pregnant women who participated in the Avon Longitudinal Study of Parents and Children (ALSPAC) [52]	Socio-demographic, fertility, reaction to pregnancy questionnaires [53]	Women with AN and AN + BN smoke more during pregnancy than healthy women. Women with AN have a higher percentage of unwanted pregnancies, 41.5% vs. 28.3% of the general population. Mothers with AN tend to have negative feelings about pregnancy, usually related to weight gain.
**Nadia Micali, 2012** [36]	Prospective longitudinal study of a birth cohort	Pregnant women who have participated in the Avon Longitudinal Study of Parents and Children (ALSPAC) [52]	Sociodemographic and anthropometric questionnaires [53] FFQ [54]	Mothers with ED consume less butter and whole milk and more legumes and soy drink compared to healthy mothers. They have a lower intake of sugars and saturated fats. Fat–protein–carbohydrate intake is the same as in unexposed mothers. They are more likely to consume >2500 mg caffeine/week. Although it is observed that the intake is adequate in terms of quality, it is not known if it is adequate in terms of quantity.
**Stephanie Zerwas, 2012** [55]	Prospective cohort study of pregnant mothers	Pregnant women who participated in the Norwegian Mother and Child Cohort Study (MoBa) [50]	MBRN—MoBa MoBa Questionnaire 1 [51] MoBa Questionnaire 4 [51]	Mothers with AN, BN, EDNOS, and BED gained weight more quickly during pregnancy and lost weight more quickly over the first six months postpartum than mothers without EDs.
**Maria Angelica Nunes, 2012** [38]	Prospective cohort study of pregnant mothers	Pregnant women who have participated in ECCAGE (The Study of Food Intake and Eating Behaviors in Pregnancy) [44,45,46]	ECCAGE Specific Questionnaire EDE-Q [47]	17.1% (*n* = 119) have binges during pregnancy. The weight gain is significantly higher in mothers who have BED (15.2 kg) than in healthy mothers (13.6 kg). It seems that it is due to the increase in these behaviors and therefore an excess in food intake.
**Knoph C, 2013** [32]	Prospective cohort study of pregnant mothers	Pregnant women who participated in the Norwegian Mother and Child Cohort Study [50]	MBRN—MoBa MoBa Questionnaire 1 [51] MoBa Questionnaire 4 [51] MoBa Questionnaire 5 [51] MoBa Questionnaire 6 [51] Hopkins Symptom Checklist-25	For the BN, 40% and 30% remitted at 18 and 36 months, respectively. For BED; 45% and 42%. It is associated with dietary patterns of increased sugar and fat intake and weight gain.
**Abigail Easter, 2013** [14]	Prospective cohort study	Pregnant women at King’s College Hospital, London	EDDS [56]	The prevalence of EDs during pregnancy is: 0.5% AN, 0.1% BN, and 1.8% BED, 0.1% used purging and 5% EDNOS. Binge eating behaviors were followed by 8.8% and 2.3% adopted compensatory behaviors regularly. 23.4% reported high weight and shape concerns.
**Stephanie C. Zerwas, 2014** [57]	Prospective cohort study	Pregnant women who participated in the Norwegian Mother and Child Cohort Study [50]	MBRN—MoBa MoBa Questionnaire 1 [51] MoBa Questionnaire 3 [51] MoBa Questionnaire 4 [51] MoBa Questionnaire 5 [51] MoBa Questionnaire 6 [51]	Mothers with AN, BN, EDNOS, and BED gained weight more quickly during pregnancy and lost weight more quickly during the six months after delivery than mothers without EDs.
**Maria Angelica Nunes, 2014** [39]	Prospective cohort study	Pregnant women who have participated in ECCAGE (The Study of Food Intake and Eating Behaviors in Pregnancy) [44,45,46]	ECCAGE Specific Questionnaire EDE-Q [47]	Excess weight gained during pregnancy and postpartum retention are associated with EDs. Self-induced binging and vomiting decreased in pregnancy and postpartum compared to pre-pregnancy. The frequency of EDs decreases during the gestation period and appears at five months.
**Cecilia Brundin Petterson, 2016** [43]	Cross-sectional study	Pregnant women and recent births from clinics in Stockholm	EDE-Q [47]	The prevalence of ED is 3% and 7.2% in pre and postpartum, respectively. Women with elevated EDE-Q values before pregnancy may experience greater conflict after delivery, finding it challenging to balance the desire to restrict caloric intake and the desire to eat.
**A.Easter, 2017** [40]	Prospective cohort study	Women who participated in the Nutrition and Stress During Pregnancy (NEST-p) study and their children [58]	SCID-I [59] EDE-Q [47] STAI [60] BDI [61] PSS [62] PRAQ-R [63]	Women with active EDs have low morning cortisol levels, suggesting that they have a significantly smaller decrease in cortisol throughout the day compared to the healthy mothers, in both adjusted and unadjusted analyses. It is therefore claimed that pregnancy in women with EDs results in dysfunction of the hypothalamic–pituitary–adrenal axis.
**Anh N. Nguyen, 2017** [42]	Estudio de cohortes	Women and their children from a Generation R Study cohort in the Netherlands [64]	293-item FFQ [54] Postnatal Breastfeeding Questionnaires	Women with a history of ED have a higher quality diet than those without any history of ED. There are no statistically significant differences between mothers with and without ED in terms of initiation and duration of breastfeeding.
**Chui Yi Chan, 2018** [65]	Prospective cohort study	Pregnant women in Hong Kong hospitals	EAT-26 [66] Anxiety Subscale of the Hospital Anxiety and Depression Scale [67] Edinburgh Postnatal Depression Scale [68] 10-item Rosenberg Self-Esteem Scale Kansas Marital Satisfaction Scale Chinese Version [69]	There is a significant non-linear relationship between time and ED, with the presence being lower during the pregnancy period and increasing in the postpartum period to levels higher than before pregnancy. Smoking is related to the presence of pre-pregnancy EDs.
**Maria Giulia Martini, 2018** [41]	Prospective cohort study of pregnant mothers and her babies	Women who participated in the Nutrition and Stress During Pregnancy (NEST-p) study and their children [58]	SCID-I [59] EDE-Q [47] IFQ [70]	60.6% of the mothers gave BM as exclusive feed at eight weeks, 64% used it partially or totally at six months, and there was no difference between mothers with and without ED. EDs are related to concerns and inappropriate feeding behaviors.
**Hunna J Watson** [71]	Prospective cohort study	Pregnant women who participated in the Norwegian Mother and Child Cohort Study [50]	MoBa Questionnaires	Higher birth weight and large-for-gestational-age in mothers were associated with BED in adjusted models. Mothers born at a lower birth weight were more likely to develop AN. Lifetime BN was not associated with perinatal factors. In this first known investigation into birth characteristics and purging disorder, no significant associations were found.

AN (anorexia nervosa); BN (bulimia nervosa); EDNOS (eating disorder not otherwise specified); BED (binge eating disorder); BM (breast milk); EDE-Q (Eating Disorder Examination Questionnaire); FFQ (Food Frequency Questionnaire); MoBa (Norwegian Mother and Child Cohort Study); MBRN (Medical Birth Registry of Norway); ECCAGE (The Study of Food Intake and Eating Behaviors in Pregnancy); ALSPAC (Avon Longitudinal Study of Parents and Children); SCID-I (Structured Clinical Interview for Axis I DSM-IV-TR Disorders); STAI (Spielberger State-Trait Anxiety Inventory); BDI (Beck Depression Inventory); PSS (Perceived Stress Scale); PRAQ-R (Pregnancy Related Anxiety Questionnaire Revised; BMI (body mass index).

## Supplementary Materials

The following are available online at https://www.mdpi.com/1010-660X/56/7/352/s1, Table S1: Analysis of methodological quality of studies in the present review. Checklist for cohort studies; Table S2: Analysis of methodological quality of studies in the present review. NICE—Checklist for cohort studies. ARHQ—Checklist for cross-sectional studies; Table S3: Analysis of the methodological quality of the studies in this review. Checklist for cohort studies.
