# Supplementary material for: Eating Disorders in Pregnant and Breastfeeding Women: A Systematic Review
Source: Medicina (Kaunas). 2020 Jul 15;56(7):352. doi: 10.3390/medicina56070352 (PMC7404459; doi:10.3390/medicina56070352)
Supplement: Supplementary file 1 [file medicina-56-00352-s001.pdf]

Appendix A

Supplementary table 1. Analysis of methodological quality of studies in the present review. Checklist for cohort studies.

	CASP - Checklist for Cohort Study / "yes" - "can,t tell" - "no" (18)						
**ITEM**	1	2	3	4	5		6	
					A	B	A	B*
**Rafael Marques Soares, 2009**	YES	YES	N/A	YES	YES. COVARIABLES	YES	YES (Questionnaires)	SI
**Cynthia M Bulik, 2009**	YES	YES	N/A	YES	YES. COVARIABLES	YES	YES (Measures, Questionnaires)	YES
**Rebecca A. Swann, 2009**	YES	YES	N/A	YES	YES. COVARIABLES	YES	YES (Measures, Questionnaires)	YES
**Leila Torgersen, 2010**	YES	YES	N/A	YES	YES. COVARIABLES	YES	YES (Measures, Questionnaires)	YES
**Anna Maria Siega-Riz PhD, RD , 2011**	YES	YES	N/A	YES	YES. COVARIABLES	YES	YES (Measures, Questionnaires)	YES
**A Easter, 2011 -**	YES	YES	N/A	YES	YES. COVARIABLES	YES	YES (Measures, Questionnaires)	YES
**Nadia Micali, 2012**	YES	YES	N/A	YES	YES. COVARIABLES	YES	YES (Measures, Questionnaires)	YES
**Stephanie Zerwas, 2012**	YES	YES	N/A	YES	YES. COVARIABLES	YES	YES (Questionnaires)	YES
**Maria Angelica Nunes, 2012**	YES	YES	N/A	YES	YES. COVARIABLES	YES	YES (Questionnaires)	YES
**Knoph C, 2013**	YES	YES	N/A	YES	YES. COVARIABLES	YES	YES (Measures, Questionnaires)	YES
**Abigail Easter, 2013**	YES	YES	N/A	YES	NO	NO	YES (Questionnaires)	YES
**Stephanie C. Zerwas, 2014**	YES	YES	N/A	YES	YES. COVARIABLES	YES	YES (Measures, Questionnaires)	YES
**Maria Angelica Nunes, 2014**	YES	YES	N/A	YES	N/A	N/A	YES (Questionnaires)	YES
**A.Easter, 2016**	YES	YES	N/A	YES	YES. COVARIABLES	YES	YES (Questionnaires)	N/A
**Anh N. Nguyen, 2017**	YES	YES	N/A	YES	YES. COVARIABLES	YES	YES (Questionnaires)	YES
**Chui Yi Chan, 2018**	YES	YES	N/A	YES	YES. COVARIABLES	YES	YES (Questionnaires)	N/A
**Maria Giulia Martini, 2017**	YES	YES	N/A	YES	YES. COVARIABLES	YES	YES (Questionnaires)	N/A

(N/A = not appear), 6B* An adequate period of time has been considered when the follow-up has been > 1 year. 1=Did the study address a clearly focused issue?, 2= Was the cohort recruited in an acceptable way?, 3= Was the exposure accurately measured to minimize bias?, 4= Was the outcome accurately measured to minimize bias?, 5= (a) Have the authors identified all important confounding factors?, (b) Have they taken account of the confounding factors in the design and/or analysis?, 6= (a) Was the follow up of subjects complete enough?, (b) Was the follow up of subjects long enough?.

Supplementary table 2. Analysis of methodological quality of studies in the present review. NICE - Checklist for cohort studies. ARHQ - Checklist for cross-sectional studies.

NICE - METHODOLOGY CHECKLIST FOR COHORT STUDY / "yes" - "no" - "unclear" - "not applicable" (18)														
	**A. Selection bias**			**B. Performance bias**			**C. Attrition bias**			**D. Detection bias**				
**ITEM**	1	2	3	1	2	3	1	2	3	1	2	3	4	5
**Rafael Marques Soares, 2009**	N/A	YES	YES	YES	N/A	N/A	YES	N/A	N/A	YES	YES	YES	N/A	N/A
**Cynthia M Bulik, 2009**	N/A	YES	YES	YES	N/A	N/A	YES	N/C	N/A	YES	YES	YES	N/A	N/A
**Rebecca A. Swann, 2009**	N/A	YES	YES	YES	N/A	N/A	YES	GROUP AN AND EDNOS VERY SMALL FOR SIGNIFICANT RESULTS	N/A	YES	YES	YES	N/A	N/A
**Leila Torgersen, 2010**	N/A	YES	YES	YES	N/A	N/A	YES	N/C	N/A	YES	YES	YES	N/A	N/A
**Anna Maria Siega-Riz PhD, RD , 2011**	N/A	YES	YES	YES	N/A	N/A	YES	GROUP AN AND EDNOS VERY SMALL AND WERE NOT ANALYZED	AN Y TCANE	YES	YES	YES	N/A	N/A
**A Easter, 2011 -**	N/A	YES	YES	YES	N/A	N/A	YES	VERY SMALL AN+BN GROUP FOR SIGNIFICANT RESULTS	N/A	YES	YES	YES	N/A	N/A
**Nadia Micali, 2012**	N/A	YES	YES	YES	N/A	N/A	YES	N/A	N/A	YES	YES	YES	N/A	N/A
**Stephanie Zerwas, 2012**	N/A	YES	YES	YES	N/A	N/A	YES	N/A	N/A	YES	YES	YES	N/A	N/A
**Maria Angelica Nunes, 2012**	N/A	YES	YES	YES	N/A	N/A	YES	N/A	N/A	YES	YES	YES	N/A	N/A
**Knoph C, 2013**	N/A	YES	YES	YES	N/A	N/A	YES	GROUP AN AND EDNOS VERY SMALL AND WERE NOT ANALYZED	AN Y TCANE	YES	YES	YES	N/A	N/A
**Abigail Easter, 2013**	NO	N/C	YES	YES	N/A	N/A	YES	N/A	N/A	YES	YES	YES	N/A	N/A
**Stephanie C. Zerwas, 2014**	N/A	YES	YES	YES	N/A	N/A	YES	N/C	N/A	YES	YES	YES	N/A	N/A
**Maria Angelica Nunes, 2014**	UNCLEAR	N/C	YES	YES	N/A	N/A	YES	NO	N/A	YES	YES	YES	N/A	N/A
**A.Easter, 2016**	N/A	YES	YES	YES	N/A	N/A	YES	N/A	N/A	N/C	YES	YES	N/A	N/A
**Anh N. Nguyen, 2017**	N/A	YES	YES	YES	N/A	N/A	YES	N/A	N/A	YES	YES	YES	N/A	N/A
**Chui Yi Chan, 2018**	N/A	YES	YES	YES	N/A	N/A	YES	N/A	N/A	N/C	YES	YES	N/A	N/A
**Maria Giulia Martini, 2017**	N/A	YES	YES	YES	N/A	N/A	YES	N/A	N/A	N/C	YES	YES	N/A	N/A

AHRQ Methodology Checklist for Cross-Sectional/Prevalence Study / "yes" - "no" - "unclear N/C"

	1	2	3	4	5	6	7	8	9	10	11
**Cecilia Brundin Petterson, 2016**	YES	YES	4 MONT HS	YES	YES	Statistical analysis of the EDE-Q	N/C	The EDE-Q questionnaire was used to assess the presence of EDs. Excluded are those with 3 or more items to be completed. Ex-poratory factor analysis was performed.	N/C	Between 92 and 98% of the mothers invited to participate	40 cases were excluded because more than 3 items were missing

N/A (not shown, not applicable), N/C (not clear). NICE: A1; The method of allocation to intervention groups was unrelated to potential confounding factors (that is, the reason for participant allocation to intervention groups is not expected to affect the outcome[s] under study). A2; Attempts were made within the design or analysis to balance the comparison groups for potential confounders. A3; The groups were comparable at baseline, including all major confounding factors. B1; The comparison groups received the same care and support apart from the intervention(s) studied. B2; Participants receiving care and support were kept 'blind' to intervention allocation. B3; Individuals administering care and support were kept 'blind' to intervention allocation. C1; All groups were followed up for an equal length of time (or analysis was adjusted to allow for differences in length of follow-up). C2; a. How many participants did not complete the intervention in each group? b. The groups were comparable for intervention completion (that is, there were no important or systematic differences between groups in terms of those who did not complete the intervention). C3= a. For how many participants in each group were no outcome data available? b. The groups were comparable with respect to the availability of outcome data (that is, there were no important or systematic differences between groups in terms of those for whom outcome data were not available). D1= The study had an appropriate length of follow-up. D2= The study used a precise definition of outcome. D3= A valid and reliable method was used to determine the outcome. D4= Investigators were kept 'blind' to participants' exposure to the intervention. D5= Investigators were kept 'blind' to other important confounding factors. **AHRQ;** 1: Define source of information (survey, record, review). 2: List inclusion and exclusion criteria for exposed and unexposed subjects (cases and controls) or refer to previous publications. 3: Indicate time period used for identifying patients. 4: Indicate whether or not subjects were consecutive if not population-based. 5: Indicate if evaluators of subjective components of study were masked to other aspects of the status of the participants. 6: Describe any assessments undertaken for quality assurance purposes (e.g., test/retest of primary outcome measurements). 7: Explain any patient exclusions from analysis. 8: Describe how confounding was assessed and/or controlled. 9: If applicable, explain how missing data were handled in the analysis. 10: Summarize patient response rates and completeness of data collection. 11: Clarify what follow-up, if any, was expected and the percentage of patients for which incomplete data or follow-up was obtained.

Supplementary table 3. Analysis of the methodological quality of the studies in this review. Checklist for cohort studies.

**NOS (Newcastle-Ottawa Scale) for Cohort Study** (18)								
	1	2	3	4	5	6	7	8
**Rafael Marques Soares, 2009**	/	*	*/	/	* inappropriate thoughts and behaviors	*/	June 2016 - September 2017	* 8.7% of mothers choose not to participate
**Cynthia M Bulik, 2009**	/	*	*/	*	** impact of eating disorders - impact on mother-children	*/	* 1999-2006	* There is no loss of subjects but only 42% of the total population of Norwegian mothers participate
**Rebecca A. Swann, 2009**	/	*	*/	*	** impact of eating disorders weight gain - children's consequences	*/	* 1999-2007	* There is no loss of subjects but only 42% of the total population of Norwegian mothers participate
**Leila Torgersen, 2010**	/	*	*/	*	* breastfeeding duration	*/	* 1999-2007	* There is no loss of subjects but only 42% of the total population of Norwegian mothers participate
**Anna Maria Siega-Riz PhD, RD , 2011**	/	*	*/	*	* Variation in weight of mothers with ED compared to healthy controls	*/	* 1999-2006	* There is no loss of subjects but only 42% of the total population of Norwegian mothers participate
**A Easter, 2011 -**	/	*	/	*	** time to conceive, unwanted pregnancies, feelings about pregnancy	/	1 April 1991 and 31 December 1992.	* There are losses (15%) but describe the reasons
**Nadia Micali, 2012**	/	*	*/	*	* quality of the diet in mothers with ED	/	1 April 1991 and 31 December 1992.	* 91.7% of the mothers were included until the end. Loss of subjects due to loss of information
**Stephanie Zerwas, 2012**	/	*	*/	*	* perception of children's temperament	*/	1999 - 2008	* There is no loss of subjects but only 38% of the total population of Norwegian mothers participate
**Maria Angelica Nunes, 2012**	/	*	*/	*	* impact of binge eating disorder during pregnancy	*/	June 2016 – September 2017	* 8.7% of mothers choose not to participate
**Knoph C, 2013**	/	*	*/	*	* remission of ED in the pregnancy-postpartum period	*	1999 - 2010	* There is no loss of subjects but only 42% of the total population of Norwegian mothers participate
**Abigail Easter, 2013**	/	*	*/	*	* status and symptoms of ED during pregnancy	*/	March 2010 and February 2012	*94% of the sample decides to participate

**Stephanie C. Zerwas, 2014**	/	*	*/	/	* weight change	*	1999 - 2008	* No loss of subjects but only 38.5% of the total population of Norwegian mothers participate
**Maria Angelica Nunes, 2014**	/	*	*/	/	* inappropriate thoughts and behaviors	*/	June 2016 - September 2017	* 8.7% of mothers choose not to participate
**A.Easter, 2016**	/	*	*/	*	* regulation of the adrenal-hypothalamic axis	*/	/	* There are no losses
**Anh N. Nguyen, 2017**	/	*	*/	*	** Quality of the mothers' diet - Duration of the LM	*/	April 2002 and January 2006	* 61% of the women who were invited to participate accepted
**Chui Yi Chan, 2018**	/	*	*/	*	*course and risk factors associated with ACTs during different periods of pregnancy	*/	/	* There's a loss, all right. Explain why.
**Maria Giulia Martini, 2017**	/	*	*/	*	* Breastfeeding in mothers with ATD	*/	/	* There are no losses

Each * corresponds to a certain criterion that is met. Good quality: 3 or 4 stars in selection domain AND 1 or 2 stars in comparability domain AND 2 or 3 stars in outcome/exposure domain . 1. Representativeness of the exposed cohort, 2. Selection of the non-exposed cohort, 3. Ascertainment of exposure, 4. Demonstration that outcome of interest was not present at start of study, 5. Comparability of cohorts on the basis of the design or analysis controlled for confounders, 6. Assessment of outcome, 7. Was follow-up long enough for outcomes to occur, 8. Adequacy of follow-up of cohorts.
